# Exercise and postprandial lipaemia: effects on peripheral vascular function, oxidative stress and gastrointestinal transit

**DOI:** 10.1186/1476-511X-6-30

**Published:** 2007-10-31

**Authors:** Miriam Clegg, Conor McClean, W Gareth Davison, H Marie Murphy, Tom Trinick, Ellie Duly, Jim McLaughlin, Mark Fogarty, Amir Shafat

**Affiliations:** 1 Department of Physical Education and Sport Sciences, University of Limerick, Limerick, Ireland; 2 Sport and Exercise Sciences Research Institute, University of Ulster, Belfast, BT37 OQB, Northern Ireland; 3 Ulster Hospital, Dundonald, BT16 1RH, Northern Ireland; 4 Nanotechnology Research Institute, University of Ulster, Belfast, BT37 OQB, Northern Ireland

## Abstract

Postprandial lipaemia may lead to an increase in oxidative stress, inducing endothelial dysfunction. Exercise can slow gastric emptying rates, moderating postprandial lipaemia. The purpose of this study was to determine if moderate exercise, prior to fat ingestion, influences gastrointestinal transit, lipaemia, oxidative stress and arterial wall function. Eight apparently healthy males (age 23.6 ± 2.8 yrs; height 181.4 ± 8.1 cm; weight 83.4 ± 16.2 kg; all data mean ± SD) participated in the randomised, crossover design, where (i) subjects ingested a high-fat meal alone (control), and (ii) ingested a high-fat meal, preceded by 1 h of moderate exercise. Pulse Wave Velocity (PWV) was examined at baseline, post-exercise, and in the postprandial period. Gastric emptying was measured using the ^13^C-octanoic acid breath test. Measures of venous blood were obtained prior to and following exercise and at 2, 4 and 6 hours post-ingestion. PWV increased (6.5 ± 1.9 m/sec) at 2 (8.9 ± 1.7 m/sec) and 4 hrs (9.0 ± 1.6 m/sec) post-ingestion in the control group (time × group interaction, P < 0.05). PWV was increased at 2 hrs post-ingestion in the control compared to the exercise trial; 8.9 ± 1.7 vs. 6.2 ± 1.5 m/sec (time × group interaction, P < 0.05). Lipid hydroperoxides increased over time (pooled exercise and control data, P < 0.05). Serum triacylglycerols were elevated postprandially (pooled exercise and control data, P < 0.05). There were no changes in gastric emptying, cholesterol, or C-reactive protein levels. These data suggest that acute exercise prior to the consumption of a high-fat meal has the potential to reduce vascular impairments.

## Background

Substantial evidence exists outlining the relationship between the postprandial state and vascular function [1,2]. It has been proposed that postprandial lipaemia (PPL) can cause endothelial dysfunction, via an oxidative stress mechanism, and that repeated episodes of PPL may promote the development of atherosclerosis [3,4]. Exercise may reduce PPL by slowing gastric emptying rates, or, increasing the removal of lipids into muscle tissue. Increased provision of fat rich nutrients to the small intestine leads to increased fat in the small intestine. Carey et al. [5] found that the efficacy of absorption of dietary triacylglycerols (TAG's) under physiological conditions in human adults is above 95%.

The resulting increased fat in the small intestine leads to greater PPL. The consequences of this are well documented in the metabolic syndrome [6-8]. However, little research exists on the effect of gastric emptying of solids following a steady state exercise bout in a health related setting. Slowing the delivery of lipids into the small intestine may reduce absorption rates and plasma concentrations of atherogenic mediators.

A large section of literature exists to highlight the effect of exercise in reducing PPL [9,10] but only when it is performed at a moderate (61% maximal oxygen uptake) but not low (31% maximal oxygen uptake) intensity [11]. Most studies use exercise intervention 12–24 hours before analysis of lipaemia status. Pertidou *et al*. [12] examined the effect of exercise immediately before a meal and although exercise did reduce PPL this did not reach significance. However, exercise in the latter study was only 45 minutes in duration and the test meal was of moderate fat content (35% from total energy) therefore not challenging lipid homeostasis significantly.

Lipid peroxidation represents an index of oxidative stress in vivo, and research examining the deleterious effects of high-fat loads on vascular function have reported concurrent increases in lipid peroxidation [13]. Bae et al. [3] highlight the relationship between a high concentration of circulating lipid, oxidative stress and endothelial function. It has been postulated that a high-fat load, may increase free radical production in the vasculature, which in turn can inactivate endothelial-derived nitric oxide (NO) causing endothelial dysfunction. Studies routinely measure endothelial function as an index of vascular integrity. Data examining the effects of PPL and arterial stiffness remain scarce, even though endothelial function and vascular (arterial) stiffness are causally associated and conceptually related [14]. It is postulated that a decrease in endothelial-derived NO bioavailability may be the critical factor in linking endothelial dysfunction and arterial stiffening [15]. Regulation of systemic vascular tone is perhaps the most documented activity of NO in human body; with research showing that endogenous NO production is closely associated with the control of blood pressure [16].

The aim of the current study was to determine if one hour of moderate exercise prior to feeding can have a protective effect on gastrointestinal transit, lipid absorption, glycaemia, oxidative stress and arterial wall function, following a high-fat meal.

## Methods

### Subject Characteristics

Following ethical approval from the local research ethics committee, 8 (n = 8) recreationally trained (participated in at least 2-hrs week^-1 ^individual or team sport) male subjects (22.9 ± 2.8 yrs; height 181.4 ± 8.1 cm; weight 83.4 ± 16.2 kg; all data mean ± SD) were recruited from the local population. Before participation all subjects completed a Health History Questionnaire to ensure that they had no medical ailments that would compromise their participation. All subjects were non-smokers and were not taking any antioxidant or lipid-lowering supplements. None of the subjects had any history of gastrointestinal disorder or suffered from gastrointestinal upset before or during the study. Complete study details including potential risks were fully explained to participants before written informed consent was obtained.

### Anthropometric measures

Upon arrival at the laboratory, measurements for body mass and stature were taken from each of the subjects. Height was measured using a freestanding stadiometer (Holtain Limited, Great Britain). Body mass was measured using scales (Seca delta, Seca, Germany).

### Experimental design

Subjects participated in a randomised, balanced, crossover study, where, (i) subjects ingested a high-fat meal alone (control), and (ii) ingested a high-fat meal, preceded by 1 h of moderate intensity exercise. Trials were separated by seven days and subjects were randomly allocated to either the control group or the exercise group for the first test and then switched to the other group for the second test, seven days later. Subjects were asked to refrain from exercise and alcohol consumption for 24 hrs before each trial. Subjects recorded their habitual dietary intake using a weighed food diary for the three days before the first trial and repeated it for the three days before the second trial. Subjects arrived at the laboratory following a standard 12 hrs overnight fast. All subjects were familiar with the exercise ergometers; to minimise diurnal variation all subjects exercised between 8 AM and 10 AM on both trials.

### High-fat meal

The high-fat physiological meal consisted of three small wholemeal pancakes, butter 0.28 g/kg body mass (Kerrygold, Ireland) bacon – 0.59 g/kg body mass (Denny smoked hickory, Ireland), mayonnaise-0.3 g/kg body mass (Hellmanns, Unilever, Ireland), mature grated cheddar cheese-0.3 g/kg body mass (St Bernard, Ireland), cooked cocktail sausages – 0.26 g/kg body mass (Ballineen fine foods, Co. Cork, Ireland). The nutritional composition of the breakfast is described in table 5. Added to the pancake mix also was 12 g of high performance inulin (Raftiline HP, Orafti, Belgium) and 100 mg of ^13^C-octanoic acid (Euriso-top, France, according to [17]). Fluid intake was limited to water; volunteers were permitted to drink 500 ml as soon as they woke up on each of the trial days and the same amount when consuming the test meal.

### Exercise protocol

For the exercise trial, subjects exercised at 60% of their maximum predicted heart rate for one hour on a cycle ergometer (Monark, Sweden). Maximum predicted heart rate was calculated according to ACSM (2007, [18]) guidelines and after the work of Martins et al. 2007 [19]. Heart rates were continuously measured using a short-range telemetry system (Polar Electro, Finland).

### Pulse wave velocity (PWV) measurement

PWV was measured at various time points; baseline, immediately post exercise, two and four hours post meal ingestion, during the experimental trials. Upon arrival at the lab, subjects were required to rest in a seated position for approximately 5 minutes. Following this the resting (baseline) PWV of each subject was measured using a sensor-based PWV device (Sensor Technology & Devices Limited, Northern Ireland) as detailed by McLaughlin et al. [20]. Pulse wave traces were calculated using the Labview program (Version 7.0). PWV was calculated (PWV (m/s) = l/Δt, where l is the distance between the two sensors and Δt corresponds to the time delay in the pulse transit time between the two sensors) following a 10 second measuring period, which was operator-initiated. PWV was measured three times for each individual subject and the mean value (m/sec) was recorded for each time point. PWV recordings were measured on the same (left) arm for all subjects, between the brachial and radial pulse sites of the arterial tree (these were identified by manual palpation) by the attachment of two polyvinylidene fluoride piezoelectric conformal and flexible sensor strips of dimension 2 cm by 5 cm. The sites were marked for each trial. The distance between the sites was measured for each subject, using a measuring tape, for both the control and exercise trials. The PWV value is based upon the time delay in the mean foot-to-foot, peak-to-peak and cross-correlational waves recorded between the two marked sites in the 10-second measuring period. All tests were conducted between the hours 08.00–15.00 to help control circadian variation. Co-efficient of variation (CV) was 2.8% for PWV.

### Blood pressure (BP)

Systemic arterial blood pressure (BP) was measured in the brachial artery using an Omran M5-I fully automatic BP monitor (Surrey, UK). Subjects were requested to rest in a supine position for five minutes before each BP measurement. BP measurements were taken twice following each of the PWV measures and the mean calculated from these two readings.

### Gastrointestinal transit

Following the 60-minutes, when heart rates had returned to below 90 bpm baseline breath hydrogen (Micromedical H_2 _meter) and breath air samples were taken. These were used for the analysis of MCTT and gastric emptying. All subjects consumed a high fat breakfast within 15 minutes. The meal contained a non-digestible substrate, inulin. Inulin causes the release of H_2 _gas into exhaled breath when it reaches the caecum and is metabolised by colonic bacteria. Similarly the addition of octanoic acid results in ^13^CO_2 _appearance in the breath; this can then be used to measure emptying from the stomach. If the meal was finished before the allocated 15 minutes the clock was reset to zero and all measurements were taken from this time onwards. ^13^CO_2 _breath samples were taken every 15 minutes for six hours. Breath hydrogen measurements were taken every 10 minutes throughout the six hours.

MCTT was defined as a consecutive increase in breath hydrogen over three consecutive readings of at least a cumulative 10 ppm [21]. Breath samples were analysed for ^13^CO_2 _using isotope ratio mass spectrometry. Data was fitted to a gastric emptying model developed by Ghoos et al. [22]. For all the data r^2 ^coefficient between the modelled and raw data was calculated and r^2 ^> 0.90. Half emptying time and lag phase were extrapolated using methods described in [22]. Latency phase and ascension time were calculated using formulas described in Schommartz et al. [23].

### Visual analogue scale (VAS)

Satiety was measured using a 150 mm VAS to detect changes in hunger, thirst, desire to eat, tiredness, fullness and cold every 30 minutes throughout the six hours. Variables thirst, tiredness and cold were used to distract subjects from analysis of their satiety status. Results are expressed as a percentage.

### Blood biochemistry

#### Blood sampling

Bloods were collected at baseline, post exercise and 2, 4 and 6 hours postprandially. Samples of blood were obtained, with minimal stasis by extracting blood from a prominent forearm vein, while subjects rested in a supine position. After immediate blood collection, antithrombotic vacutainers containing potassium ethylenediaminetetraacetic acid (K3 EDTA) and Lithium heparin were placed on ice, whilst the serum separating clot activator tubes were allowed to clot at room temperature (for 15 minutes) before centrifugation began at 3500 rpm for 5 mins at 4°C. Plasma and serum were removed and transferred to 1.5 ml plastic vials and were stored at -70°C before subsequent biochemical analysis. Post-exercise blood samples were corrected for plasma volume shifts using the method of Dill & Costill (1974) [24].

### Serum C-reactive protein (CRP)

CRP samples were assayed on an Aeroset TM analyser (Abbott Labs, USA) using a CRP high sensitivity assay kit (Randox Laboratories Ltd, Northern Ireland). A 5 ml sample of venous blood was collected into a SST tube and spun at 3000 rpm at 4°C for 10 minutes to allow for separation. The sample was combined with a buffer and an anti-CRP coated latex. The formation of the antibody-antigen complex during the reaction results in an increase in turbidity, the extent of which is measured as the amount of light absorbed at 550 nm. CV was 1.82% at 2.18 Mg.L^-1^ and 1.85% at 4.93 .Mg.L^-1^

### Plasma glucose levels

Glucose levels were determined by immobilised enzyme membrane method in conjunction with a Clark electrode on a YSI 2300 analyser (Yellow Springs, USA). The principle of the test involves measuring electrode flow from a steady state H_2_O_2 _concentration, which is proportional to the concentration glucose.

### Blood lipids

Total cholesterol, TAG's and HDL cholesterol (HDL-C) levels were measured by enzyme assay kits, using the Aeroset TM analyser (Abbott Labs, USA). For total cholesterol, samples were centrifuged for 10 minutes at 3000 rpm at room temperature. Samples were reacted with cholesterol esterase (CE) and then cholesterol oxidase (CO) resulting in the formation of a chromophore, which was quantitated at 500 nm. For TAG's, samples were hydrolysed by lipase, the resultant glycerol fractions are phosphorylated and then oxidised. Samples are then reacted with peroxidase and 4-aminoantipyrine and 4-chlorophenol and the colour produced relates to the TAG content of the sample. For HDL-C, samples were centrifuged at 3600 rpm for 10 minutes to separate. A polyanion was added to assist with complexing cholesterol subfractions. The second reagent released only HDL-C allowing it to react with CE and CO, in the presence of chromagen to produce colour. Estimates of LDL-C concentration were calculated using the Friedewald formula [25]. CV was less than 2.5% for all blood lipid indices.

### Measurement of serum lipid hydroperoxides (LOOH)

Serum LOOHs were measured using the FOX-1 assay [26]. Standard solutions were incubated for 30 minutes with the FOX-1 reagent. Preparation of the FOX-1 reagent involves the addition of the following ingredients: 100 μM.L^-1^ Xylenol orange, 250 μM.L^-1 ^Ammonium ferrous sulphate, 100 mM.L^-1 ^Sorbitol and 25 mM.L^-1 ^of sulphuric acid (H_2_SO_4_). After thawing, 50 μl of each serum aliquot was added to 950 μl of FOX-1 reagent. Samples were then vortexed and left to incubate for 30 minutes at room temperature in a dark room. The absorbance of the supernatant was then read at 560 nm against the standard curve for the concentration range 0–5 μM.L^-1^. CV was 4.6% at 0.57 μM.L^-1^.

### Statistical analysis

Statistical analysis was performed using the SPSS social statistics package-version 11.0 (Surrey, UK). All data, except VAS, were checked for normal distribution and equal variance. These data were analysed using a repeated measures 2-way analysis of variance (ANOVA), with one between (group) and one within (time) subject factor. For a significant interaction effect, within subject factors were analysed using Bonferroni-corrected paired samples t-test. Between subject differences were analysed using a one-way ANOVA with a posteriori Tukey Honestly Significant Difference (HSD) test. Visual analogue scale data were transformed natural log and analysed as above. Gastrointestinal transit was compared using Wilcoxon signed ranks test. The alpha level was established at P < 0.05. Prospective calculations of power were performed according to the equations of Altman [27]. Retrospective calculations of power were carried out (using SPSS) for specific variables to determine the suitability of the sample size employed. All data are expressed as mean ± SD unless otherwise stated.

## Results

### Vascular function

Table 1 indicates that following ingestion of the high-fat test meal, mean PWV increased at 2 h, and 4 h respectively, post ingestion in the control trial (time × group interaction, P < 0.05), compared to baseline values. Mean PWV also increased at 2 h, and 4 h respectively, post ingestion in the control trial compared to pre ingestion values (time × group interaction, P < 0.05). There was no difference in mean PWV values at the baseline and pre ingestion time points in the control trial. In the exercise trial, no changes in mean PWV were observed at each of the time points when compared to baseline values respectively (time × group interaction, P > 0.05). In the exercise trial mean PWV values at 3 h post ingestion were lower than the corresponding time point in the control trial; 6.2 ± 1.5 m/sec vs. 8.9 ± 1.7 m/sec (time × group interaction, P < 0.05). Retrospective calculation of power = 0.95.

**Table 1:** Comparison of PWV prior to and following ingestion of high-fat meal

**Time**	**Mean PWV (m/sec ± S.D)**	
	
	**Control**	**Exercise**
Baseline	6.48 ± 1.91	7.11 ± 1.84
Pre ingestion	6.56 ± 1.56	6.47 ± 1.90
Two hours post ingestion	8.90 ± 1.67*	6.15 ± 1.30†
Four hours post ingestion	8.99 ± 1.61 *	6.85 ± 1.10†

*P = < 0.05 vs. baseline control trial; † P = < 0.05 vs. 2 h post ingestion control

### Oxidative stress indices

There was no change in mean serum LOOH levels between baseline and pre ingestion intervals in both trials (time × group interaction, P > 0.05). Mean serum LOOH increased at 2 h and 4 h post ingestion in the control trial compared to baseline values (time × group interaction, P < 0.05). Mean serum LOOH were elevated at 2 h post ingestion in the exercise trial compared to baseline exercise levels (time × group interaction, P < 0.05). There was no difference between mean serum LOOH levels at 4 h post ingestion and baseline levels in the exercise trial (time × group interaction, P > 0.05). LOOH increased over time (pooled exercise and control data, P < 0.05). Retrospective calculation of power = 0.73.

### Cardiovascular risk indices

There was a main effect for time for TAG levels (Table 2.), with TAG levels increasing over time (pooled control and exercise data, P < 0.05) but there were no changes in mean TAG levels between the groups (time × group interaction, P > 0.05). There were no changes in mean serum total cholesterol, estimated serum LDL-C levels, serum HDL-C or serum CRP either within or between the groups (time × group interaction, P > 0.05). Retrospective calculation of power for TAC, LDL, HDL, total cholesterol and CRP = 0.29, 0.11, 0.22, 0.15 and 0.09 respectively.

**Table 2:** Biochemical markers following ingestion of high-fat meal & exercise

			***Mean Interval (± S.D)***		
**Variable**	**Baseline**	**Pre-ingestion**	**2 h-post ingestion**	**4 h-post ingestion**	**6-h post ingestion**
*Control*					
Total cholesterol (mmol/L)	3.74 ± 0.99	3.07 ± 1.2	3.63 ± 1.23	3.81 ± 0.42	3.75 ± 0.62
LDL cholesterol (mmol/L)	2.21 ± 0.67	1.88 ± 0.48	1.83 ± 0.57	1.91 ± 0.39	2.02 ± 0.3
HDL cholesterol (mmol/L)	1.15 ± 0.35	0.92 ± 0.65	1.16 ± 0.43	1.27 ± 0.55	1.22 ± 0.45
triglycerides (mmol/L)	0.68 ± 0.23	0.55 ± 0.26	1.14 ± 0.22	1.40 ± 0.70	0.87 ± 0.19
CRP (mg/L)	1.24 ± 1.28	0.71 ± 0.39	0.92 ± 0.65	0.95 ± 0.87	1.19 ± 1.25
glucose (mmol/L)	5.06 ± 0.38	4.9 ± 0.28	4.51 ± 0.24	5.06 ± 0.18	N/A
LOOH (μM)	1.52 ± 0.39	2.26 ± 1.10	2.13 ± 0.91*	2.46 ± 1.20*	1.77 ± 0.8
Systolic BP (mmHg)	128 ± 3.2	129.8 ± 8.6	132.9 ± 6.8	130.5 ± 5.0	N/A
Diastolic BP (mmHg)	76.6 ± 6.1	76.2 ± 9.1	75.7 ± 5.9	73.5 ± 7.9	N/A
					
*Exercise*					
Total cholesterol (mmol/L)	3.93 ± 0.91	3.63 ± 1.02	4.06 ± 0.92	3.73 ± 1.11	3.58 ± 1.25
LDL cholesterol (mmol/L)	2.37 ± 0.64	2.25 ± 0.71	2.08 ± 0.49	2.01 ± 0.54	2.09 ± 0.66
HDL cholesterol (mmol/L)	1.16 ± 0.33	1.07 ± 0.26	1.18 ± 0.36	1.04 ± 0.48	1.00 ± 0.47
triglycerides (mmol/L)	0.68 ± 0.24	0.51 ± 0.23	1.18 ± 0.24	0.93 ± 0.46	0.66 ± 0.27
CRP (mg/L)	1.28 ± 0.85	1.08 ± 0.83	1.26 ± 0.88	1.24 ± 0.83	1.26 ± 0.88
glucose (mmol/L)	4.95 ± 0.29	4.59 ± 0.26†	4.76 ± 0.41	4.98 ± 0.27	N/A
LOOH (μM)	1.19 ± 0.25	0.97 ± 0.36	2.28 ± 0.90†	2.13 ± 1.19	1.44 ± 0.57
Systolic BP (mmHg)	131.4 ± 4.5	130.1 ± 4.7	131.8 ± 4.9	135 ± 6.3	N/A
Diastolic BP (mmHg)	76.8 ± 6.8	73.9 ± 7.8	70.9 ± 4.9	77.6 ± 5.6	N/A

* P = < 0.05 vs. baseline control trial; † P = < 0.05 vs. baseline exercise trial

***Re: ***triglycerides n = 7 subjects as an outlier was removed

### Blood glucose

Mean glucose levels decreased immediately pre ingestion in the exercise trial compared to baseline values (time × group interaction, P < 0.05). There was no change in glucose levels between the same time points in the control trial (time × group interaction, P > 0.05). Retrospective calculation of power = 0.68.

### Gastrointestinal transit

There were no differences between exercise and rest conditions for mouth to caecum transit time or gastric emptying half time, lag phase, latency phase and ascension time (Table 3; P > 0.05). Gastric emptying half time following exercise decreased by 12.6% in comparison to the control condition. Similarly MCTT decreased by 22.2% following the exercise condition compared to control.

**Table 3:** Gastric emptying half time and mouth to caecum transit time of high fat meal

**Gastrointestinal transit**		
	
	**Control**	**Exercise**
Gastric emptying half time (mins)	238 ± 137	208 ± 98
Gastric emptying lag phase (mins)	70 ± 29	69 ± 22
Gastric emptying latency phase (mins)	43 ± 29	58 ± 39
Gastric emptying ascension time (mins)	291 ± 139	236 ± 83
MCTT (mins)	126 ± 56	98 ± 44

***Re: ***MCTT n = 7 subjects

### Visual analogue scale

There was a change in all parameters except cold over the 6-hour period (Table 4; pooled control and exercise data, P < 0.05), where hunger and desire to eat increased, fullness decreased and subjects felt thirstier and more tired. There were differences between exercise and control trials for satiety parameters; hunger and desire to eat (time × group interaction, P > 0.05) but not fullness.

**Table 4:** Visual analogue scale results average over 6 hours (13 time points) expressed as median and interquartile range (n = 8) following a high fat meal. 100% indicates "not at all", 0% indicates "very".

**Variable**	**Median visual analogue scale ratings (% (interquartile range))**	
	
	**Control**	**Exercise**
Hunger	46.9 (34.3–52.0)	46.5 (29.4–54.8)*
Thirst	42.9 (20.3–50.8)	33.2 (16.5–51.8)
Desire to eat	43.0 (30.9–58.3)	39.1 (30.4–47.9)*
Tiredness	62.7 (53.7–73.0)	58.9 (43.3–72.5)
Fullness	64.6 (50.6–72.5)	63.5 (53.7–71.1)
Cold	72.6 (64.4–78.9)	73.5 (58.4–81.1)

* P = < 0.05 vs. control trial.

**Table 5:** Nutritional content of test meal based on 70 kg male

	**Weight**	**Energy Content**	**Fat**	**Protein**	**Carbohydrate**
	
	**g**	**KJ (kcal)**	**Nutrient content (g)**		
Meal	249	3331 (796)	60.4	30.3	35.9
			**% from energy**		
			68.2	15.2	16.9

### Blood pressure

There were no changes in either mean systolic or mean diastolic BP throughout the duration of both trials (time × group interaction, P > 0.05).

## Discussion

There were no changes observed in gastric emptying or MCTT within this study. Average gastric emptying half-time was long in duration, as expected from a high-fat test meal [28]. One hour exercise before the consumption of a high-fat meal did not alter either measure of gastrointestinal transit. It has previously been reported that chronic exercise, and the associated high energy dietary intake, accelerates oro-caecal transit [29]. One hour of moderate intensity exercise was not sufficient to alter gastric emptying or lipaemia and a longer time period, higher exercise intensity or repeated bouts may be necessary. These results suggest that nutrient delivery rate into the small intestine and into the blood were similar in both resting and exercise conditions. Because there were no differences in glycaemia, lipaemia and cholesterolaemia between the two conditions, it is likely that any changes in arterial PWV are due to other modulators of arterial wall function.

A main finding of the current study is that a bout of moderate intensity aerobic exercise, performed immediately prior to meal ingestion, prevented the increase in brachial-radial PWV observed in the control trial (no exercise) throughout the postprandial period. When the TAG data from both trials was pooled, there was a main effect for time, whereby TAG levels increased compared to baseline over the course of the trials. The authors believe that the ingestion of the high-fat meal is the most likely cause of this increase in postprandial TAG levels. In the control trial, mean PWV increased at 2 h and 4 h postprandially. However, in the exercise group mean PWV values were no different to baseline levels at 2 h and 4 h post-ingestion. Interestingly, the beneficial changes in PWV values occurred independent of changes in blood pressure, which supports existing work that has detailed improvements in arterial function without any corresponding blood pressure changes [15].

The results from the control trial compliment the work of others [30] who have shown that the ingestion of a single high-fat meal can significantly impair vascular function. Furthermore, the results suggest an augmented oxidative stress manifested by an increase in LOOH levels in the postprandial period. It has been postulated that the impairments in vascular function observed following the ingestion of a high-fat meal are the result of an oxidative stress mechanism [31].

We have previously demonstrated the benefits of a single bout of moderate intensity exercise on oxidative stress levels and PWV following a high-fat meal [1]. The mechanisms through which exercise could counteract the deleterious effects of a high-fat meal may be multifactorial. Many studies advocate exercise training as a method to improve endothelial dysfunction in man [32]. Exercise increases blood flow to the coronary circulation and active skeletal tissue. Exercise training may facilitate arterial adaptations leading to an improvement in endothelial function [33]. The increase in blood flow (shear stress) associated with exercise is thought to up-regulate endothelial nitric oxide synthase (eNOS) expression [34] which could potentially result in an increase in NO bioavailability. The effects of acute exercise on vascular function have been less readily examined, however, acute increases in shear stress have not only been shown to increase eNOS mRNA expression but increase the capacity of endothelial cells to release NO [35]. Wilkinson et al. [36] have shown improvements in PWV through increased stimulation of NO by exogenous acetylcholine, thus the exercise-induced improvements in PWV observed in this study could be attributed to an increase in NO availability brought about by a shear stress mechanism.

A bout of moderate intensity aerobic exercise, as demonstrated in the current study seems to attenuate the increase in lipid peroxidation. Whilst LOOH levels increased at 2 h postprandially (compared to baseline) in both groups, there was no difference in LOOH levels at 4 h postprandially, compared to baseline, in the exercise trial. LOOH levels at the same time-point were significantly greater than baseline levels in the control (no exercise) trial. This would represent a decrease in oxidative stress levels as a result of exercise intervention and consequently the ameliorating effects of exercise on PWV could thus be due to the decrease in oxidative stress markers. It has been postulated that exercise may perpetuate an increase in ambient SOD levels [37], which has the ability to scavenge superoxide radicals and consequently prevent the increase in LOOH levels following the high-fat meal, as observed in the control trial. Furthermore, an increase in SOD levels could potentially increase NO bioavailability, which as previously suggested may result in improvements in vascular function. It may be of benefit when conducting any further research in this area to measure NO activity. NO_X _levels reflect the circulating nitrate/nitrite concentrations, which are stable end products of NO [38]. It has been shown that both exhaustive exercise [39] and moderate intensity training programmes [40] have accentuating effects on NO_X _levels but little research exists examining the effect of acute exercise on NO_X _levels. It may also be of benefit to measure SOD levels in any further research. Acute moderate exercise has been shown to yield improvements in SOD function and confer protection against oxidative damage [41]. Indeed data from our laboratory has observed a negative correlation between brachial-radial PWV and blood SOD levels (r^2 ^= -0.383, P < 0.05 [1]).

## Conclusion

Although the benefits of exercise are widely known to decrease the risk of cardiovascular disease [42], debate remains over the optimum intensity and duration of exercise that would promote such benefits. The current study highlights the benefits to PWV of a single session of moderate intensity exercise performed prior to the ingestion of a high-fat meal. It seems likely that these benefits could possibly be the result of a reduction in oxidative stress levels attributed to the exercise bout irrespective of gastrointestinal transit or lipaemia.

## Competing interests

The author(s) declare that they have no competing interests.

## Authors' contributions

MC, CM, AS and GWD have made substantial contributions to conception and design, or acquisition of data, analysis and interpretation of data, have been involved in drafting the manuscript, revising it critically for important intellectual content. MF, TT, ED, MH, JM have made substantial contributions to acquisition of data and analysis and interpretation of data. All authors read and approved the final manuscript.

## References

[B1] McClean CM, McLauhglin JAD, Burke G, Murphy MH, Trinick T, Duly E, Davison GW (2007). The effect of acute aerobic exercise on pulse wave velocity and oxidative stress following postprandial hypertriglyceridemia in healthy men. Eur J Appl Physiol.

[B2] Nitenberg A, Cosson E, Pham I (2006). Postprandial endothelial dysfunction: role of glucose, lipids and insulin. Diabetes Metab.

[B3] Bae JH, Bassenge E, Kim KB, Kim YN, Kim KS, Lee HJ, Moon KC, Lee MS, Park KY, Schwemmer M (2001). Postprandial hypertriglyceridemia impairs endothelial function by enhanced oxidant stress. Atherosclerosis.

[B4] Ceriello A, Taboga C, Tonutti L, Quagliaro L, Piconi L, Bais B, Da Ros R, Motz E (2002). Evidence for an independent and cumulative effect of postprandial hypertriglyceridemia and hyperglycemia on endothelial dysfunction and oxidative stress generation: effects of short- and long-term simvastatin treatment. Circulation.

[B5] Carey MC, Small DM, Bliss CM (1983). Lipid digestion and absorption. Annu Rev Physiol.

[B6] Reaven GM (1988). Banting lecture 1988: Role of insulin resistance in human disease. Diabetes.

[B7] Kaplan NM (1989). The deadly quartet. Upper-body obesity, glucose intolerance, hypertriglyceridemia, and hypertension. Arch Intern Med.

[B8] Foster DW (1989). Insulin resistance – a secret killer?. N Engl J Med.

[B9] Gill JM, Mees GP, Frayn KN, Hardman AE (2001). Moderate exercise, postprandial lipaemia and triacylglycerol clearance. Eur J Clin Invest.

[B10] Herd SL, Kiens B, Boobis LH, Hardman AE (2001). Moderate exercise, postprandial lipemia, and skeletal muscle lipoprotein lipase activity. Metabolism.

[B11] Tsetsonis NV, Hardman AE (1996). Effects of low and moderate intensity treadmill walking on postprandial lipaemia in healthy young adults. Eur J Appl Physiol Occup Physiol.

[B12] Petridou A, Gerkos N, Kolifa M, Nikolaidis MG, Simos D, Mougios V (2004). Effect of exercise performed immediately before a meal of moderate fat content on postprandial lipaemia. Br J Nutr.

[B13] van Oostrom AJ, Sijmonsma TP, Verseyden C, Jansen EH, de Koning EJ, Rabelink TJ, Castro Cabezas M (2003). Postprandial recruitment of neutrophils may contribute to endothelial dysfunction. J Lipid Res.

[B14] Safar M, Chamiot-Clerc P, Dagher G, Renaud JF (2001). Pulse pressure, endothelium function, and arterial stiffness in spontaneously hypertensive rats. Hypertension.

[B15] Kinlay S, Creager MA, Fukumoto M, Hikita H, Fang JC, Selwyn AP, Ganz P (2001). Endothelium-derived nitric oxide regulates arterial elasticity in human arteries in vivo. Hypertension.

[B16] Gewaltig MT, Kojda G (2002). Vasoprotection by nitric oxide: mechanisms and therapeutic potential. Cardiovasc Res.

[B17] Maes BD, Ghoos YF, Geypens BJ, Hiele MI, Rutgeerts PJ (1993). Relation between gastric emptying rate and energy intake in children compared with adults. Gut.

[B18] American College of Sports Medicine (2007). ACSM's Guidelines for Exercise Testing and Prescription.

[B19] Martins C, Morgan LM, Bloom SR, Robertson MD (2007). Effects of exercise on gut peptides, energy intake and appetite. J Endocrinol.

[B20] McLaughlin J, McNeill M, Braun B, McCormack PD (2003). Piezoelectric sensor determination of arterial pulse wave velocity. Physiological Measurement.

[B21] Bond JH, Levitt MD, Prentiss R (1975). Investigation of small bowel transit time in man utilizing pulmonary hydrogen (H_2_) measurements. J Lab Clin Med.

[B22] Ghoos YF, Maes BD, Geypens BJ, Mys G, Hiele MI, Rutgeerts PJ, Vantrappen G (1993). Measurement of gastric emptying rate of solids by means of a carbon-labelled octanoic acid breath test. Gastroenterology.

[B23] Schommartz B, Ziegler D, Schadewaldt P (1998). Significance of diagnostic parameters in [^13^C]octanoic acid gastric emptying breath tests. Isotopes Environ Health Stud.

[B24] Dill DB, Costill DL (1974). Calculation of percentage changes in volumes of blood, plasma, and red cells in dehydration. J Appl Physiol.

[B25] Friedewald WT, Levy RI, Fredrickson DS (1972). Estimation of the concentration of low-density lipoprotein cholesterol in plasma, without use of the preparative ultracentrifuge. Clin Chem.

[B26] Wolff SP (1994). Ferrous ion oxidation in presence of ferric ion indicator xylenol orange for measurement of hydroperoxides. Methods Enzymol.

[B27] Altman DG (1980). Statistics and ethics in medical research: III How large a sample?. Br Med J.

[B28] Edelbroek M, Horowitz M, Maddox A, Bellen J (1992). Gastric emptying and intragastric distribution of oil in the presence of a liquid or a solid meal. J Nucl Med.

[B29] Harris A, Lindeman AK, Martin BJ (1991). Rapid orocecal transit in chronically active persons with high energy intake. J Appl Physiol.

[B30] Gaenzer H, Sturm W, Neumayr G, Kirchmair R, Ebenbichler C, Ritsch A, Föger B, Weiss G, Patsch JR (2001). Pronounced postprandial lipemia impairs endothelium-dependent dilation of the brachial artery in men. Cardiovasc Res.

[B31] Tsai WC, Li YH, Lin CC, Chao TH, Chen JH (2004). Effects of oxidative stress on endothelial function after a high-fat meal. Clin Sci.

[B32] Walther C, Gielen S, Hambrecht R (2004). The effect of exercise training on endothelial function in cardiovascular disease in humans. Exerc Sport Sci Rev.

[B33] Moyna NM, Thompson PD (2004). The effect of physical activity on endothelial function in man. Acta Physiol Scand.

[B34] Hambrecht R, Adams V, Erbs S, Linke A, Krankel N, Shu Y, Baither Y, Gielen S, Thiele H, Gummert JF, Mohr FW, Schuler G (2003). Regular physical activity improves endothelial function in patients with coronary artery disease by increasing phosphorylation of endothelial nitric oxide synthase. Circulation.

[B35] Uematsu M, Ohara Y, Navas JP, Nishida K, Murphy TJ, Alexander RW, Nerem RM, Harrison DG (1995). Regulation of endothelial cell nitric oxide synthase mRNA expression by shear stress. Am J Physiol.

[B36] Wilkinson IB, Qasem A, McEniery CM, Webb DJ, Avolio AP, Cockcroft JR (2002). Nitric oxide regulates local arterial distensibility in vivo. Circulation.

[B37] Fukai T, Folz RJ, Landmesser U, Harrison DG (2002). Extracellular superoxide dismutase and cardiovascular disease. Cardiovasc Res.

[B38] Di Massimo C, Scarpelli P, Penco M, Tozzi-Ciancarelli MG (2004). Possible involvement of plasma antioxidant defences in training-associated decrease of platelet responsiveness in humans. Eur J Appl Physiol.

[B39] Ashton T, Young IS, Davison GW, Rowlands CC, McEneny J, Van Blerk C, Jones E, Peters JR, Jackson SK (2003). Exercise-induced endotoxemia: the effect of ascorbic acid supplementation. Free Radic Biol Med.

[B40] Maeda S, Tanabe T, Otsuki T, Sugawara J, Iemitsu M, Miyauchi T, Kuno S, Ajisaka R, Matsuda M (2004). Moderate regular exercise increases basal production of nitric oxide in elderly women. Hypertens Res.

[B41] Wang JS, Lee T, Chow SE (2006). Role of exercise intensities in oxidized low-density lipoprotein-mediated redox status of monocyte in men. J Appl Physiol.

[B42] Thompson PD, Buchner D, Pina IL, Balady GJ, Williams MA, Marcus BH, Berra K, Blair SN, Costa F, Franklin B, Fletcher GF, Gordon NF, Pate RR, Rodriguez BL, Yancey AK, Wenger NK (2003). Exercise and physical activity in the prevention and treatment of atherosclerotic cardiovascular disease: a statement from the Council on Clinical Cardiology (Subcommittee on Exercise, Rehabilitation, and Prevention) and the Council on Nutrition, Physical Activity, and Metabolism (Subcommittee on Physical Activity). Circulation.

